# Evaluations of aqueous humor protein markers in different types of glaucoma

**DOI:** 10.1097/MD.0000000000031048

**Published:** 2022-10-14

**Authors:** Haiyan Chen, Gang Zheng, Huijie Chen, Lu Li, Zhuojun Xu, Li Xu

**Affiliations:** a Hainan Eye Hospital and Key Laboratory of Ophthalmology, Zhongshan Ophthalmic Center, Sun Yat-Sen University, Haikou, Hainan Province, China; b Department of Ophthalmology, Zhujiang Hospital, Southern Medical University, Guangzhou, China.

**Keywords:** aqueous humor, glaucoma, pathogenesis, protein marker

## Abstract

To compare the concentrations of protein markers in aqueous humor (AH) of patients with primary open-angle glaucoma (POAG), chronic angle-closure glaucoma (CACG), acute primary angle closure (APAC), and cataract without glaucoma as the control group.

AH samples were collected at the beginning of surgery from 82 eyes of 82 patients who were divided into POAG (n = 23), CACG (n = 21), APAC (n = 19), and cataract groups (n = 19). The expression levels of interferon-gamma (IFN-γ), interleukin-2 (IL-2), interleukin-6 (IL-6), interleukin-8 (IL-8), interleukin-17A (IL-17A), lymphotoxin-alpha (LT-α), monocyte chemotactic protein-1 (MCP-1), matrix metalloproteinase-2 (MMP-2), brain derived neurotrophic factor (BDNF), basic fibroblast growth factor (bFGF), platelet-derived growth factor-AA (PDGF-AA), vascular endothelial growth factor (VEGF), tissue inhibitor of metalloproteinases-1 (TIMP-1), and tumor necrosis factor-alpha (TNF-α) in AH were detected using a microsphere-based immunoassay.

The AH levels of TNF-α, MMP-2, MCP-1, IFN-γ, and TIMP-1 in the APAC and CACG groups were significantly higher than those in control eyes. Additionally, the AH levels of interleukin-6 (IL-6) and VEGF in the APAC group were significantly higher than those in the control group (CG). The interleukin-8 (IL-8) levels in patients with POAG were significantly higher than those in control eyes, whereas the LT-α levels were significantly lower than those in control eyes. IL-6 levels were significantly correlated with the coefficient of variation (CV), whereas IL-6 levels were significantly negatively correlated with the frequency of hexagonal cells (HEX) and corneal endothelial cell density (CD).

The levels of TNF-α, MMP-2, MCP-1, IFN-γ, TIMP-1, IL-6, IL-8, VEGF, and LT-α were different among the three types of glaucoma. These different types of glaucoma may be caused by various pathogeneses, which opens avenues for further investigation into the pathogenesis of glaucoma and discoveries new targets and pathways for the treatment of glaucoma.

## 1. Introduction

Glaucoma is the leading cause of irreversible blindness worldwide. Based on the anatomical shape of the anterior chamber angle, glaucoma can be divided into open-angle glaucoma and angle-closure glaucoma. Worldwide, the number of people with glaucoma is expected to increase to 111.8 million by 2040, disproportionately affecting those residing in Asia and Africa.^[1]^ In primary angle-closure glaucoma (PACG), the outflow pathway of the aqueous humor (AH) is obstructed anatomically by the apposition of the iris,^[2]^ whereas in patients with primary open-angle glaucoma (POAG), ocular hypertension results from increased AH outflow resistance in the trabecular meshwork (TM), which is likely associated with increased local deposition of the extracellular matrix and stiffening of the tissue.^[3–5]^

The exact mechanism underlying glaucoma pathogenesis remains unknown. PACG and POAG is associated with increased intraocular pressure (IOP), optic nerve damage, and progressive vision loss, but the molecular mechanism that underpins retinal ganglion neuropathy in PACG and POAG remains poorly understood. To better understand the pathogenesis of PACG and POAG, we performed a comprehensive proteomic analysis of AH samples from PACG, POAG, and matched controls to investigate the pathogenic alterations in AH composition.

## 2. Materials and Methods

### 2.1. Patient and group

The study protocol was approved by the Research Ethics Committee (2018-006) of Hainan Eye Hospital, Zhongshan Ophthalmic Center, Sun Yat-sen University, and informed consent was obtained from all subjects. All patients underwent careful and detailed ophthalmic evaluations, including anterior chamber angle, visual field, optic disc morphology, and IOP.

Participants were divided into four groups: POAG, chronic angle-closure glaucoma (CACG), and acute primary angle closure (APAC), cataract patients without glaucoma served as the control group (CG).

The enrollment and grouping criteria were listed as follows:

POAG was defined as glaucomatous optic neuropathy (GON) on fundoscopic examination, characteristic visual field defects on the Humphrey Field Analyzer, open anterior chamber angle on gonioscopy, and elevated IOP > 21 mm Hg on the Goldmann applanation tonometer.^[6]^ GON was diagnosed if cup/disc asymmetry >0.2 between eyes, rim thinning, notching, excavation, or retinal nerve fiber layer defects were present. The characteristic glaucomatous visual field defects were Bjerrum, Seidel, paracentral scotoma, or nasal step on Humphrey visual fields, with clusters of three or more adjacent points depressed by >5 dB or two adjacent points depressed by >10 dB.^[7,8]^

CACG was diagnosed as follows: GON, characteristic visual field defects, an occludable angle, and elevated IOP of > 21 mm Hg without a history of acute attack. An occludable angle was defined as one in which the posterior trabecular meshwork (usually pigmented) was visible for less than 90° of the angle circumference without indentation.^[9]^ For each patient with CACG, the eye with the greater mean deviation (MD) on visual field testing was defined as the observed eye for AH collection.

APAC was defined according to the following consolidated criteria: presence of at least two of the following symptoms: ocular or periocular pain, nausea and/or vomiting, headache, and an antecedent history of intermittent blurring of vision with haloes; presence of an occluded angle on gonioscopy and initial IOP greater than 21 mm Hg; and presence of at least three of the following signs: conjunctival injection, corneal epithelial edema, mid-dilated unreactive pupil, and shallow anterior chamber.^[10,11]^

Patients with CACG and APAC were scheduled for anterior chamber paracentesis, peripheral iridectomy, or trabeculectomy, and their fellow eyes with primary angle closure suspect were scheduled for prophylactic peripheral iridectomy. Patients with secondary angle closure, such as those with lens-induced glaucoma, neovascular glaucoma, or uveitis, were excluded.

The CG consisted of cataract patients without glaucomatous optic neuropathy and no history of IOP exceeding 21 mm Hg. These patients underwent routine phacoemulsification surgery with an intraocular lens implantation.

The exclusion criteria were as follows: eyes with other visually significant ocular pathologies (e.g. uveitis, optic neuritis, diabetic retinopathy, retinal vascular occlusions, macular degeneration, etc); active ocular infection or inflammation, such as active allergic conjunctivitis, active trachoma, endophthalmitis, etc; use of anti-inflammatory eye drops within one week prior to examination; use of medication that could affect visual sensitivity; and history of ocular surgery within three months of examination.

### 2.2. Sample collection and biomarker assessments

Approximately 150 μL of AH was obtained through an anterior chamber puncture procedure before cataract or glaucoma surgery, using a 27-gauge needle attached to a microsyringe. During AH collection, the needle was carefully positioned to avoid touching the corneal endothelium, iris, or the lens. The absence of blood contamination was visually confirmed. The samples were placed in Eppendorf tubes and stored at −80 °C until analysis.

At the end of the study, all AH samples were analyzed for the concentrations of 14 proteins using a microsphere-based immunoassay (Luminex, Austin, TX, USA) with four multiplex panels and a lysozyme assay.

### 2.3. Statistical analysis

Statistical analyses were performed using SPSS version 15.0, AH protein marker concentrations among the 4 groups were compared using analysis of variance with Tukey post hoc test. Correlations between AH protein marker concentrations and clinical parameters, including preoperative IOP, axial length (AL), corneal endothelial cell density (CD), frequency of hexagonal cells (HEX), coefficient of variation (CV), and retinal nerve fiber layer (RNFL), were evaluated and analyzed using Pearson correlation coefficient.

## 3. Results

### 3.1. Demographic data of study participants

The demographics and clinical features of the CACG, POAG, APAC, and CG are shown in Table 1.

**Table 1:** Demographics and clinical characteristics of the chronic primary angle-closure glaucoma group, the primary open angle glaucoma group, the acute primary angle closure group and control group.

Characteristics	CG	CACG	POAG	APAC	*P* values (CG vs CACG)	*P* values (CG vs POAG)	*P* values (CG vs APAC)	*P* values (CACG vs POAG)	*P* values (CACG vs APAC)	*P* values (POAG vs APAC)
Number of eyes	19	21	23	19						
Mean age ± SD	63.33 ± 16.79	65.86 ± 11.03	64 ± 8.939	61.11 ± 9.116	.5729	.8688	.6154	.5412	.1482	.3068
IOP (mm Hg) ± SD	16.87 ± 3.714	30.6 ± 13.62	26.24 ± 11.37	32.17 ± 11.58	<.001	<.01	<.0001	.2714	.7063	.1159
AL (mm) ± SD	23.52 ± 1.047	22.78 ± 0.8132	23.89 ± 1.765	22.59 ± 1.115	.0199	.4391	.0195	.0173	.5702	.0209
PSD (dB) ± SD	/	6.764 ± 3.582	5.713 ± 3.734	5.324 ± 2.562	/	/	/	.4383	.2676	.7684
VFI (%) ± SD	/	33.8 ± 33.06	36.73 ± 39.47	63.18 ± 32.32	/	/	/	.827	.0332	.0817
MD (dB) ± SD	/	-22.51 ± 9.117	-21.32 ± 11.4	-16.97 ± 8.835	/	/	/	.0817	.1343	.3033
CD (mm^2^) ± SD	2466 ± 283.2	2439 ± 288.8	2354 ± 308.5	2205 ± 881.6	.3033	.2328	.2354	.3785	.3011	.3033
HEX (%) ± SD	52.74 ± 9.011	52.06 ± 6.44	51.23 ± 13.5	41.75 ± 16.84	.7939	.6813	.0264	.8127	.0251	.0826
CV (%) ± SD	36.44 ± 4.788	36.62 ± 4.945	36.02 ± 4.569	43.14 ± 12.51	.2328	.7759	.0415	.693	.0534	.0208
The nasal quadrant RNFL (μm) ± SD	68.28 ± 21.03	43.55 ± 23.39	29.43 ± 27.51	77.69 ± 31.78	.0016	.0016	.3285	.0851	.0012	<.0001
The temporal quadrant RNFL (μm) ± SD	75.33 ± 11.78	49.6 ± 22.96	37 ± 27.58	87.77 ± 26.48	.0001	<.0001	.0868	.121	.0001	<.0001
The upper quadrant RNFL (μm) ± SD	124.5 ± 27.4	56.3 ± 31.19	45.05 ± 32.58	143 ± 44.99	<.0001	<.0001	.1715	.2659	<.0001	<.0001
The lower quadrant RNFL (μm) ± SD	125.7 ± 18.38	57.45 ± 32.11	42.1 ± 41.15	145.5 ± 52.87	<.0001	<.0001	.1516	.1921	<.0001	<.0001

AL = Axial length, APAC = acute primary angle closure, CACG = chronic angle-closure glaucoma, CD = corneal endothelial cell density, CG = control group, CV = coefficient of variation, HEX = frequency of hexagonal cells, IOP = intraocular pressure, MD = mean deviation, POAG = primary open-angle glaucoma, PSD = pattern standard deviation, RNFL = retinal nerve fiber layer, SD = standard deviation, VFI = visual field index.

A total of 21 patients with CACG, 19 with APAC, 23 with POAG, and 19 with age-related cataracts who fulfilled the inclusion criteria were enrolled in the study. All surgeries were performed uneventfully. There were no significant differences in sex and mean age among the four groups.

As shown in Table 1, the mean AL was significantly shorter in the CACG and APAC groups than that in the CG and POAG groups. The average CV level was significantly higher in the APAC group than that in the POAG and CG groups. The average HEX level was significantly higher in the CACG group than that in the APAC group.

### 3.2. Levels of AH protein markers in 3 types of glaucoma

**Figure 1.**
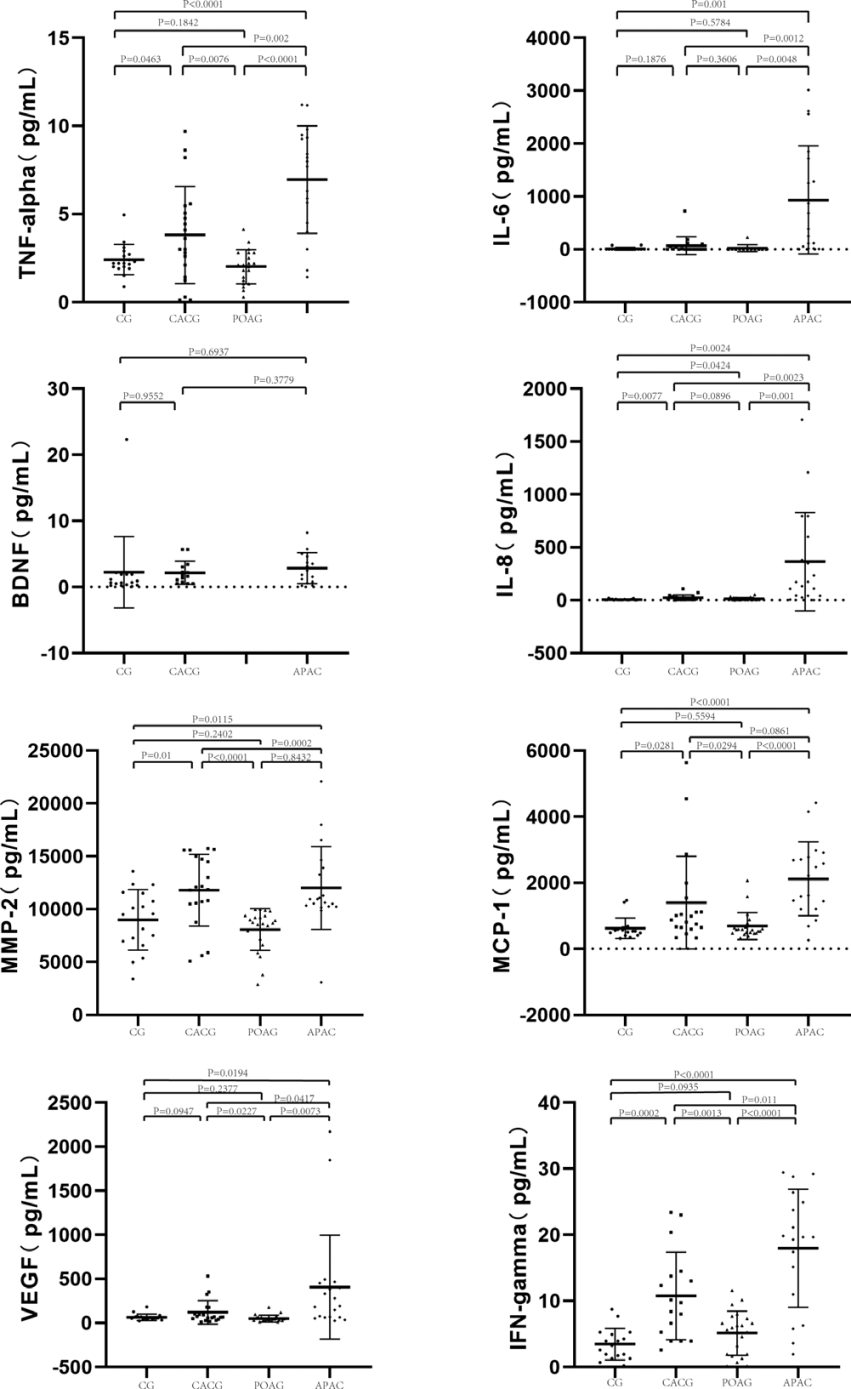
Levels of AH protein markers in the CACG, POAG, APAC group and the CG. Bars designate the means with 95% confidence intervals. *P* values were calculated by 1-way analysis of variance and multiple comparisons.AH = aqueous humor, APAC = acute primary angle closure, CACG = chronic angle-closure glaucoma, CG = control group, POAG = primary open-angle glaucoma.

**Figure 2.**
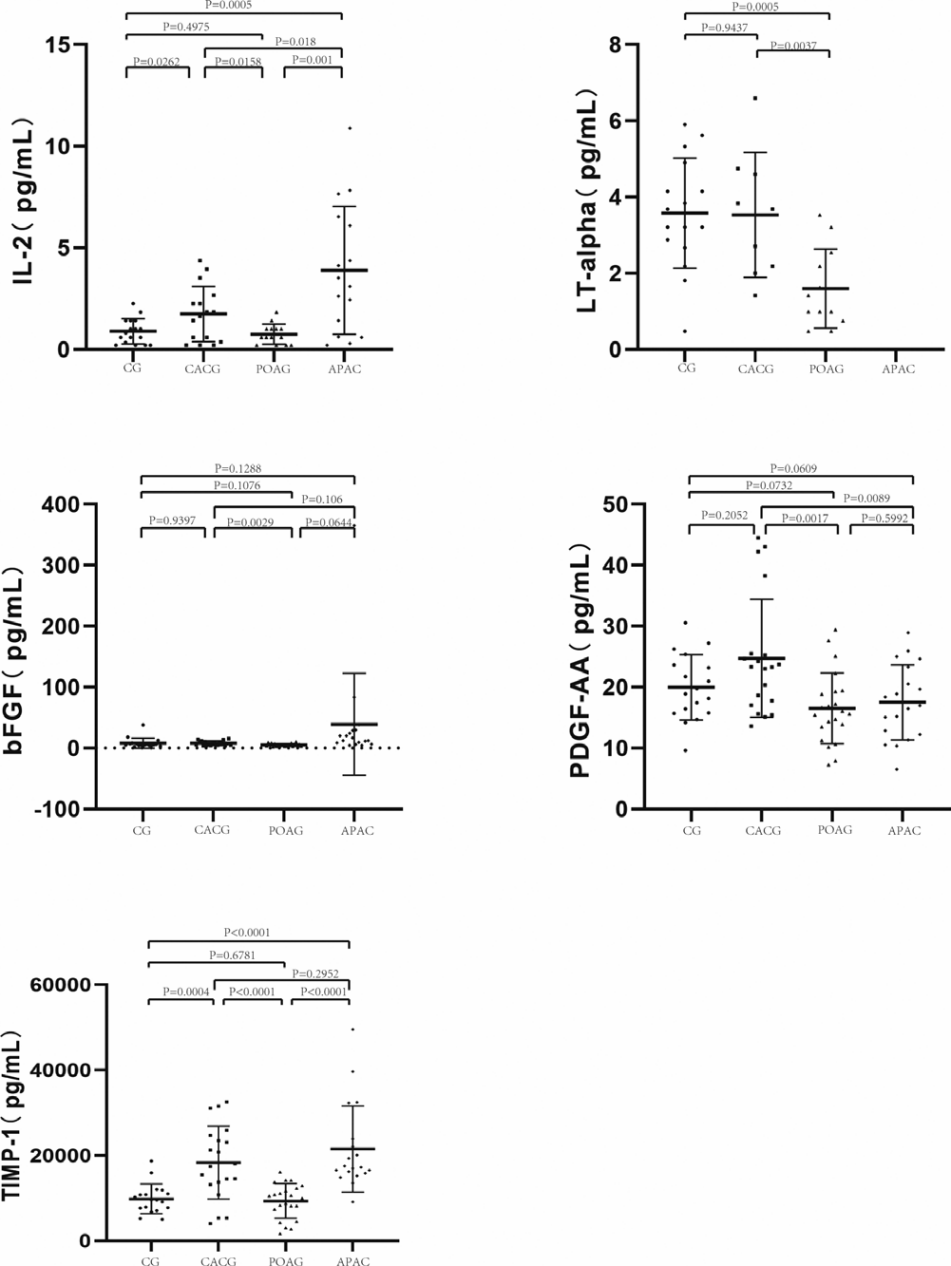
Levels of AH protein markers in the CACG, POAG, APAC group and the CG. Bars designate the means with 95% confidence intervals. *P* values were calculated by 1-way analysis of variance and multiple comparisons. AH = aqueous humor, APAC = acute primary angle closure, CACG = chronic angle-closure glaucoma, CG = control group, POAG = primary open-angle glaucoma.

The AH levels of the protein markers are listed in Table 2. The differences in the AH levels of the protein markers among the four groups are shown in Figures 1 and 2.

**Table 2:** Levels of aqueous humor protein markers in the chronic primary angle-closure glaucoma group, the primary open angle glaucoma group, the acute primary angle closure group and control group.

Cytokine (pg/mL)	CG	CACG	POAG	APAC	*P* value (CG vs CACG)	*P* values (CG vs POAG)	*P* values (CG vs APAC)	*P* values (CACG vs POAG)	*P* values (CACG vs APAC)	*P* values (POAG vs APAC)
TNF-alpha	2.413 ± 0.857	3.815 ± 2.760	2.014 ± 0.9687	6.956 ± 3.046	.0463	.1842	<.0001	.0076	.002	<.0001
IL-6	10.83 ± 26.76	69.16 ± 171.1	20.88 ± 64.75	933.3 ± 1024	.1876	.5784	.0011	.3606	.0012	.0048
BDNF	2.25 ± 5.408	2.164 ± 1.747	/	2.852 ± 2.32	.9552	/	.6937	/	.3779	
IL-8	6.22 ± 5.181	24.08 ± 26.37	13.08 ± 13.01	365 ± 465.7	.0077	.0424	.0024	.0896	.0023	.001
MMP-2	8985 ± 2866	11779 ± 3403	8068 ± 1982	12013 ± 3921	.01	.2402	.0115	<.0001	.8432	.0002
MCP-1	623.6 ± 313.7	1400 ± 1406	692.5 ± 407	2119 ± 1117	.0281	.5594	<.0001	.0294	.0861	<.0001
VEGF	64.35 ± 37.12	120.6 ± 134.2	49.63 ± 39.77	406.2 ± 590	.0947	.2377	.0194	.0227	.0417	.0073
IFN-gamma	3.452 ± 2.392	10.75 ± 6.624	5.104 ± 3.347	17.95 ± 8.928	.0002	.0935	<.0001	.0013	.011	<.0001
IL-17A	/	/	/	/	/	/	/	/	/	/
IL-2	0.8894 ± 0.6162	1.743 ± 1.368	0.7493 ± 0.495	3.894 ± 3.155	.0262	.4975	.0005	.0158	.018	.001
LT-alpha	3.576 ± 1.449	3.531 ± 1.638	1.598 ± 1.041	/	.9437	.0005	/	/	/	/
bFGF	8.274 ± 8.314	8.119 ± 3.58	5.24 ± 2.183	39.07 ± 83.52	.9397	.1076	.1288	.0029	.106	.0644
PDGF-AA	19.97 ± 5.369	24.73 ± 9.674	16.53 ± 5.801	17.52 ± 6.147	.2052	.0732	.0609	.0017	.0089	.5992
TIMP-1	9847 ± 3458	18347 ± 8547	9341 ± 4064	21520 ± 10084	.0004	.6781	<.0001	<.0001	.2952	<.0001

APAC = acute primary angle closure, bFGF = basic fibroblast growth factor, BDNF = brain derived neurotrophic factor, CACG = chronic angle-closure glaucoma, CG = control group, IFN-gamma = interferon-gamma, IL-2 = interleukin-2, IL-6 = interleukin-6, IL-8 = interleukin-8, IL-17A = interleukin-17A, LT-alpha = lymphotoxin-alpha, MCP-1 = monocyte chemotactic protein-1, MMP-2 = matrix metalloproteinase-2, PDGF-AA = platelet-derived growth factor-AA, POAG = primary open-angle glaucoma, TIMP-1 = tissue inhibitor of metalloproteinases-1, TNF-alpha = tumor necrosis factor-alpha, VEGF = vascular endothelial growth factor.

#### 3.2.1. Each glaucoma group compared with the CG.

The levels of tumor necrosis factor-alpha (TNF-α) (*P* = .0463), IL-8 (*P* = .0077), matrix metalloproteinase-2 (MMP-2) (*P* < .01), interferon-gamma (IFN-γ) (*P* = .0002), monocyte chemotactic protein-1 (MCP-1) (*P* = .0281), and tissue inhibitor of metalloproteinases-1 (TIMP-1) (*P* = .0004) were significantly elevated in the AH of patients with CACG, whereas IL-8 concentrations in the AH of patients with POAG were significantly higher (*P* = .0424). The AH levels of lymphotoxin-alpha (LT-α) (*P* = .0005) in the POAG group were significantly lower than those in the CG. The AH levels of TNF-α (*P* < .0001), IL-8 (*P* = .0024), IL-6 (*P* = .0011), MMP-2 (*P* = .0115), IFN-γ (*P* < .0001), MCP-1 (*P* < .0001), vascular endothelial growth factor (VEGF) (*P* = .0194), and tissue inhibitor of metalloproteinases-1 (TIMP-1) (*P* < .0001) were significantly higher in the APAC group than those in the CG.

#### 3.2.2. Compared between the CACG group and the POAG group.

AH levels of TNF-α (*P* = .0076), MMP-2 (*P* < .0001), VEGF (*P* = .0227), IFN-γ (*P* = .0013), MCP-1 (*P* = .0294), basic fibroblast growth factor (bFGF) (*P* = .0029), platelet-derived growth factor-AA (PDGF-AA) (*P* = .0017), TIMP-1 (*P* < .0001), LT-α (*P* = .037) in the CACG group were significantly greater than in the POAG group.

#### 3.2.3. Compared between the CACG group and the APAC group.

The mean concentrations of TNF-α (*P* = .02), IL-6 (*P* = .0012), IL-8 (*P* = .0023), VEGF (*P* = .0417), and IFN-γ (*P* = .011) were significantly lower in the CACG than those in the APAC group. The mean PDGF-AA concentration (*P* = .0089) in the CACG group was significantly higher than that in the APAC group.

#### 3.2.4. Compared between the POAG group and the APAC group.

The mean concentrations of TNF-α (*P* < .001), IL-6 (*P* = .0048), IL-8 (*P* < .001), MMP-2 (*P* = .0002), MCP-1 (*P* < .0001), VEGF (*P* = .0073), TIMP-1 (*P* < .0001), and IFN-γ (*P* < .0001) were significantly lower in the POAG group than those in the APAC group.

Interleukin-17A (IL-17) levels were lower than the detection values in all subjects. LT-α was lower than the detection value in the APAC group and brain-derived neurotrophic factor (BDNF) was lower than the detection value in the POAG group.

### 3.3. Correlations between AH protein markers and parameters of all subjects

Additionally, we investigated the correlations between AH protein markers and characteristic parameters, including preoperative IOP, AL, corneal endothelial CD, frequency of HEX, CV, RNFL, MD, PSD, and visual field index (VFI) in all subjects.

**Figure 3.**
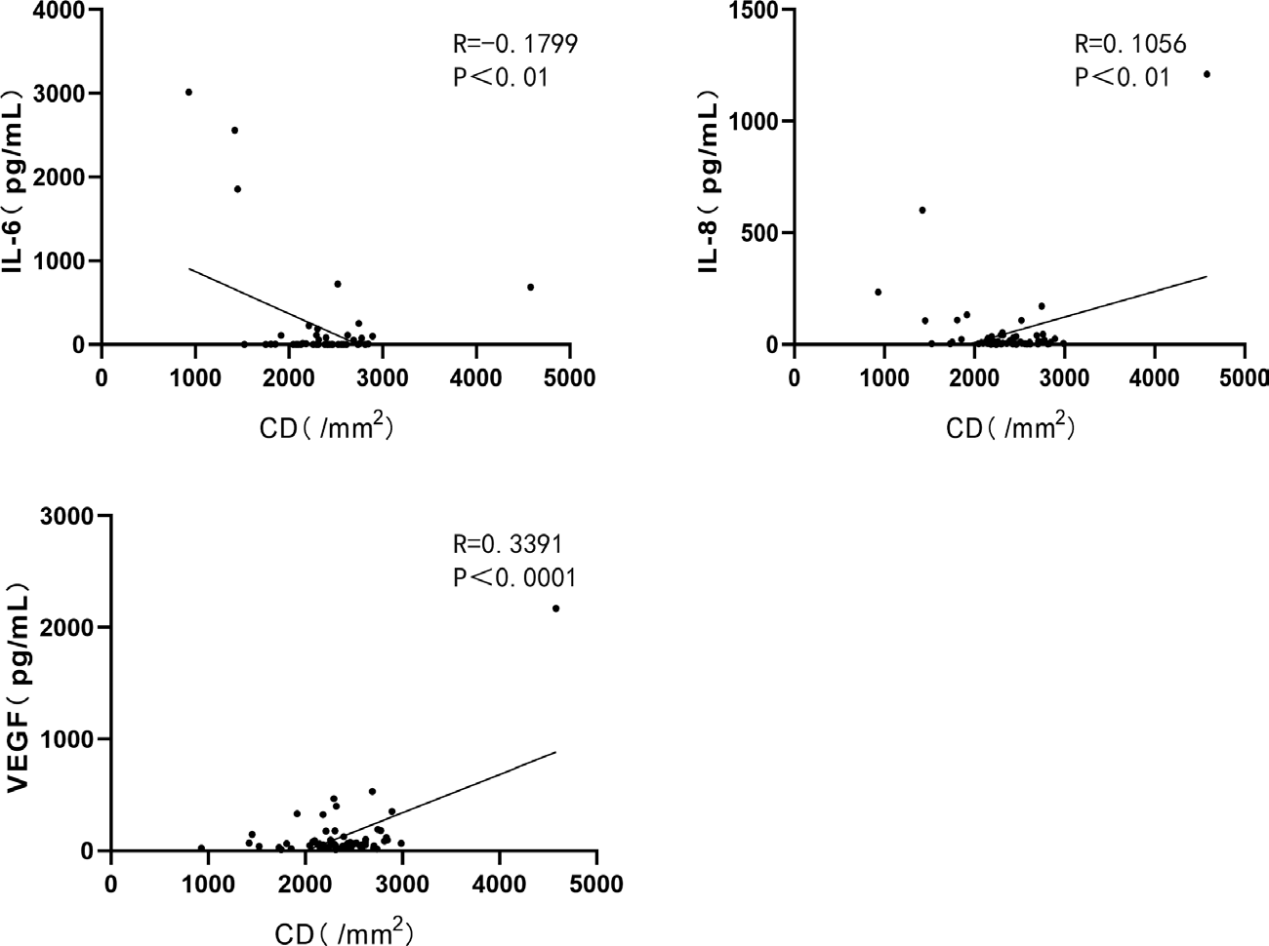
Correlation between AH protein maker and CD in the all subjects. CD with IL-6: *R* = 0.1799, *P* < .01; CD with IL-8: *R* = 0.1056, *P* < .01; VEGF: *R* = 0.3391, *P* < .0001. AH = aqueous humor, CD = corneal endothelial cell density, IL-6 = interleukin-6, IL-8 = interleukin-8, VEGF = vascular endothelial growth factor.

**Figure 4.**
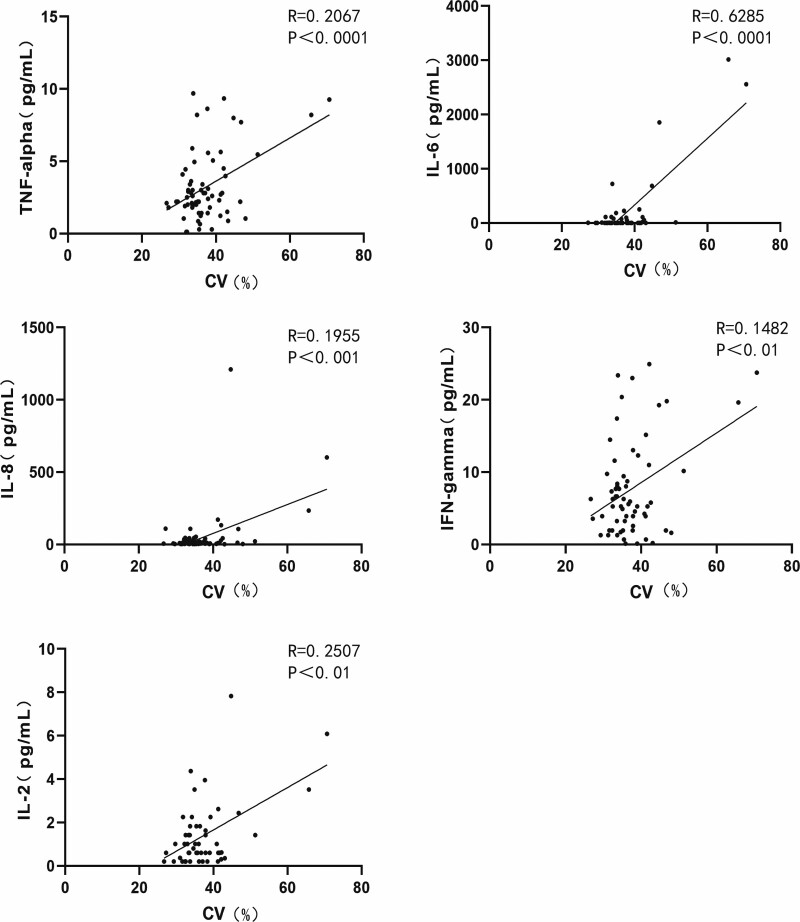
Correlation between AH protein maker and CV in the all subjects. CV with TNF-α: *R* = 0.2067, *P* < .0001; CV with IL-6: *R* = 0.6285, *P* < .0001; CV with IL-8: *R* = 0.1955, *P* < .001; CV with IFN-γ: *R* = 0.1482, *P* < .01; CV with IL-2: *R* = 0.2507, *P* < .01. AH = aqueous humor, CV = coefficient of variation, IFN-γ = interferon-gamma, IL-2 = interleukin-2, IL-6 = interleukin-6, IL-8 = interleukin-8, TNF-α = tumor necrosis factor-alpha.

**Figure 5.**
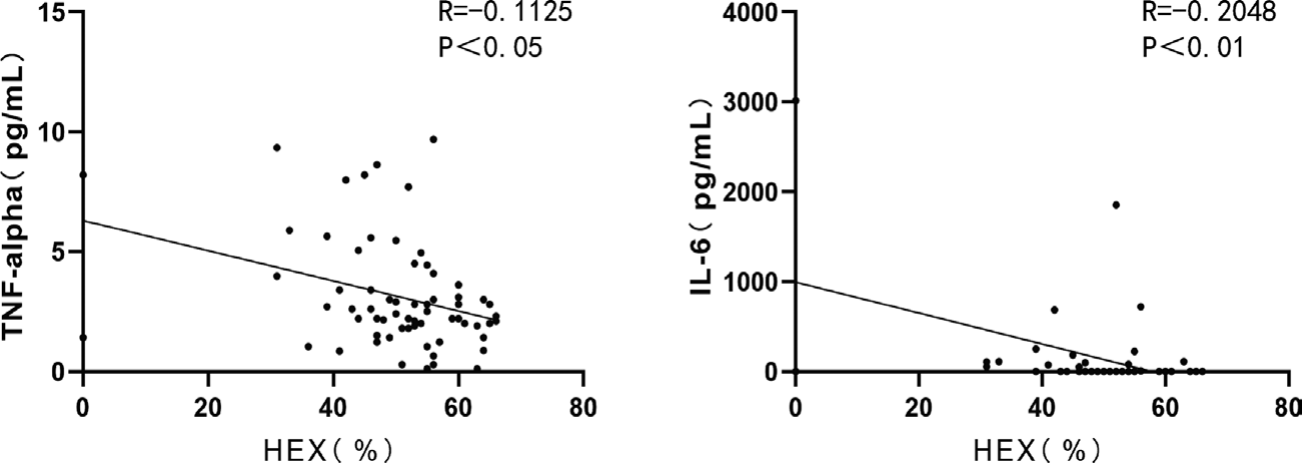
Correlation between AH protein maker and HEX in the all subjects. HEX with TNF-α: *R* = 0.1125, *P* < .05; HEX with IL-6: *R* = 0.2048, *P* < .01. AH = aqueous humor, HEX = frequency of hexagonal cells, IL-6 = interleukin-6, TNF-α = tumor necrosis factor-alpha.

**Figure 6.**
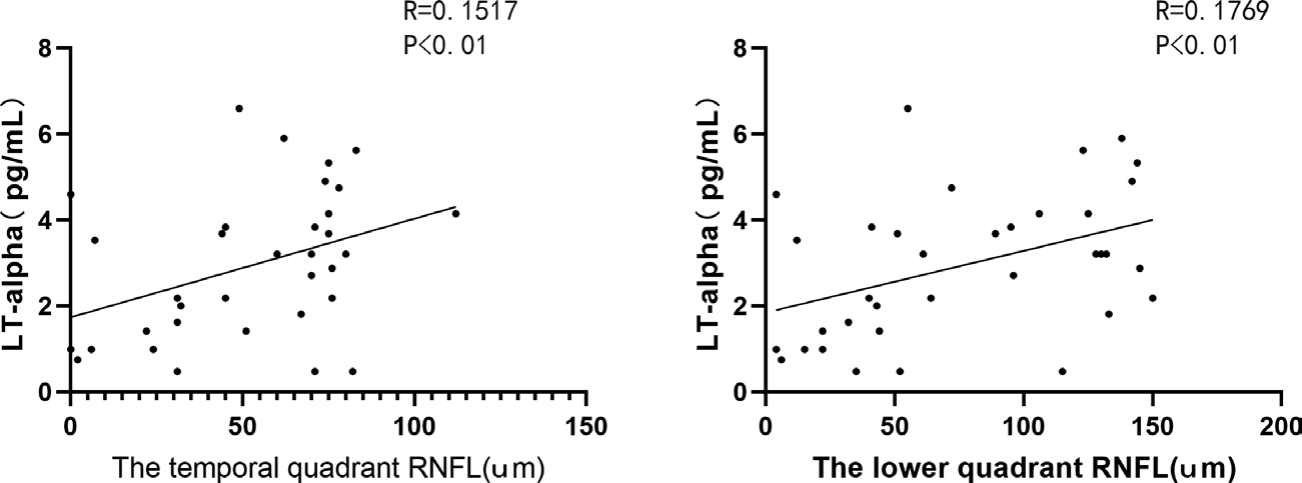
Correlation between AH protein maker and RNFL in the all subjects. the temporal quadrant RNFL with LT-α: *R* = 0.1517, *P* < .01; the lower quadrant RNFL with LT-α: *R* = 0.1769, *P* < .01. AH = aqueous humor, LT-α = lymphotoxin-alpha, RNFL = retinal nerve fiber layer.

**Figure 7.**
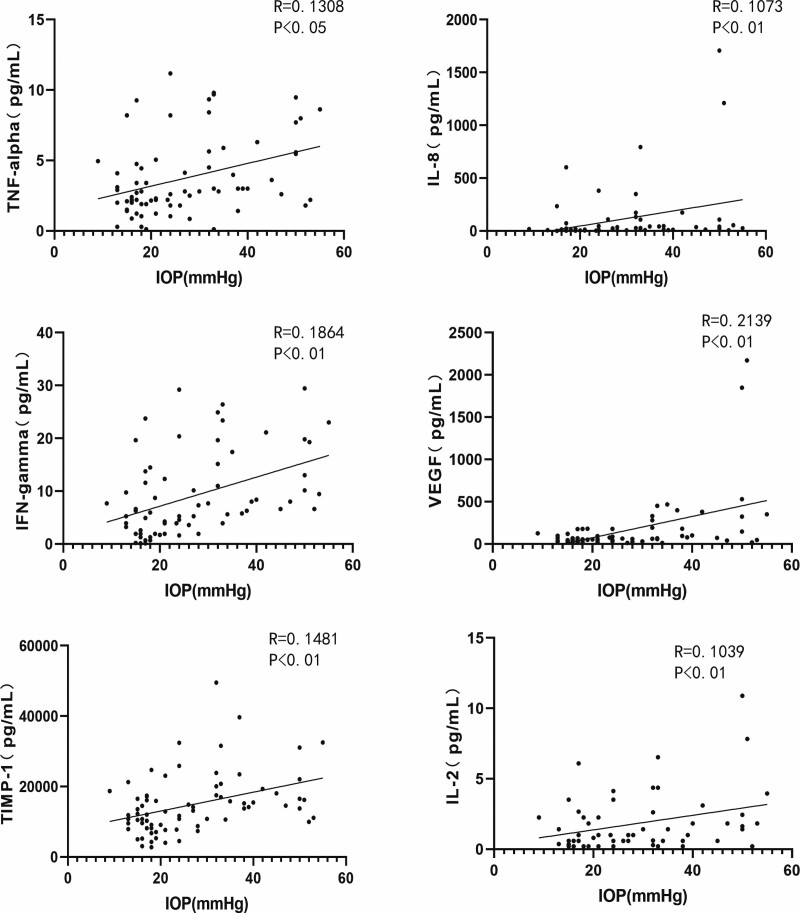
Correlation between AH protein maker and IOP in the all subjects. IOP with TNF-α: *R* = 0.1308, *P* < .05; IOP with IL-8: *R* = 0.1073, *P* < .01; IOP with IFN-γ: *R* = 0.1864, *P* < .01; IOP with VEGF: *R* = 0.2139, *P* < .01: IOP with TIMP-1: *R* = 0.1481, *P* < .01; IOP with IL-2: *R* = 0.1039, *P* < .01. AH = aqueous humor, IFN-γ = interferon-gamma, IL-2 = interleukin-2, IL-8 = interleukin-8, IOP = intraocular pressure, TIMP-1 = tissue inhibitor of metalloproteinases-1, TNF-α = tumor necrosis factor-alpha, VEGF = vascular endothelial growth factor.

CD levels significantly correlated with the expression levels of IL-6, IL-8, and VEGF. (Figure 3. CD with IL-6: *R* = 0.1799, *P* < .01; CD with IL-8: *R* = 0.1056, *P* < .01; CD with VEGF: *R* = 0.3391, *P* < .0001.) The CV levels were positively correlated with the expression levels of TNF-α, IL-6, IL-8, IFN-γ, and interleukin-2 (IL-2). (Figure 4. CV with TNF-α: *R* = 0.2067, *P* < .0001; CV with IL-6: *R* = 0.6285, *P* < .0001; CV with IL-8: *R* = 0.1955, *P* < .001; CV with IFN-γ: *R* = 0.1482, *P* < .01; CV with IL-2: *R* = 0.2507, *P* < .01) HEX levels were significantly correlated with TNF-α and IL-6 levels. (Figure 5. HEX with TNF-α: *R* = 0.1125, *P* < .05; HEX with IL-6: *R* = 0.2048, *P* < .01.) The temporal and lower quadrant RNFL were significantly correlated with LT-α levels (Figure 6. The temporal quadrant RNFL with LT-α: *R* = 0.1517, *P* < .01; lower quadrant RNFL with LT-α: *R* = 0.1769, *P* < .01.) Preoperative IOP significantly correlated with TNF-α, IL-8, IFN-γ, VEGF, TIMP-1, and IL-2 levels. (Figure 7. IOP with TNF-α: *R* = 0.1308, *P* < .05; IOP with IL-8: *R* = 0.1073, *P* < .01; IOP with IFN-γ: *R* = 0.1864, *P* < .01; IOP with VEGF: *R* = 0.2139, *P* < .01; IOP with TIMP-1: *R* = 0.1481, *P* < .01; IOP with IL-2: *R* = 0.1039, *P* < .01.)

### 3.4. Correlations between the concentrations of AH protein markers of all subjects

**Figure 8.**
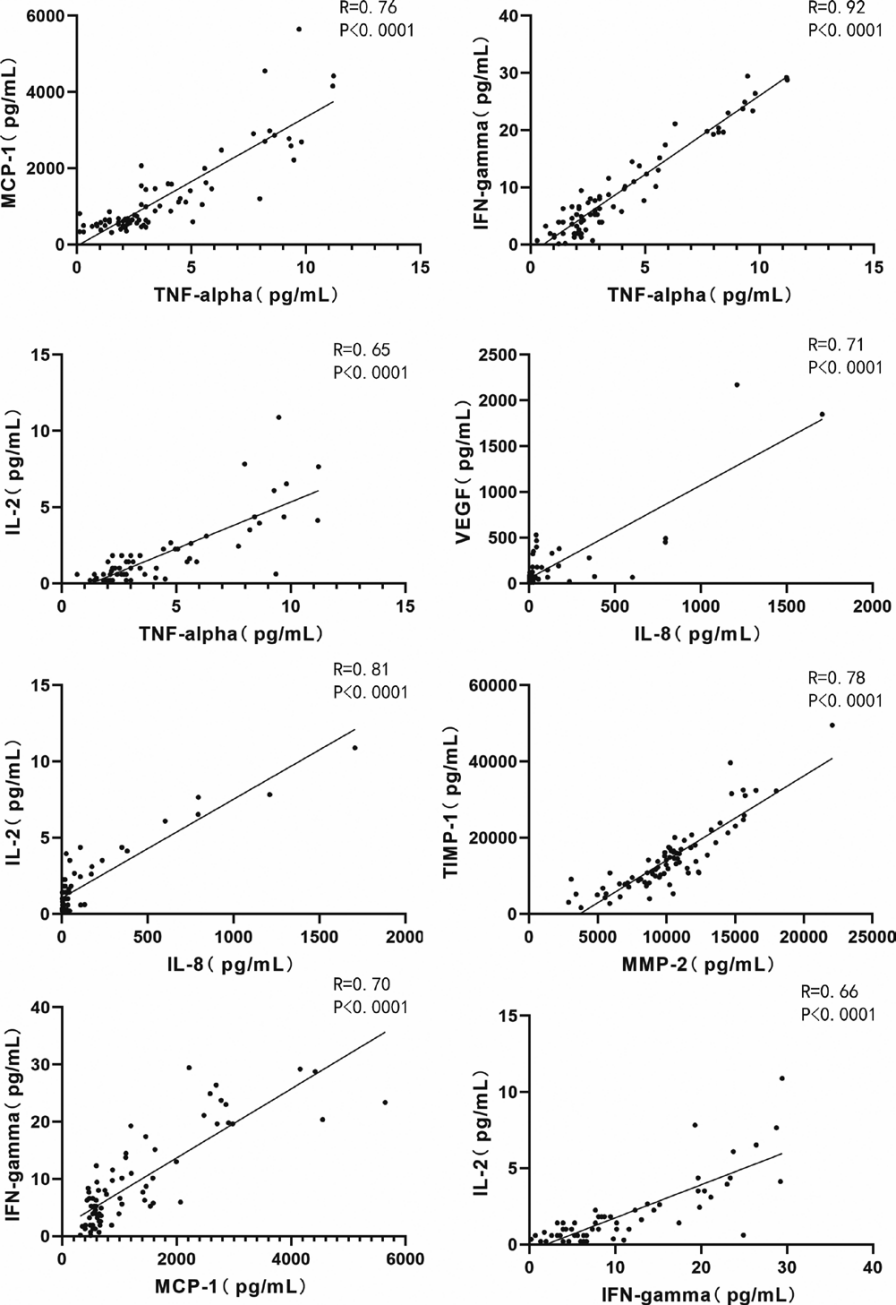
Correlation between protein markers of all subjects. TNF-α with MCP-1: *R* = 0.76, *P* < .0001; TNF-α with IFN-γ: *R* = 0.92, *P* < .0001; TNF-α with IL-2: *R* = 0.65, *P* < .0001; IL-8 with VEGF: *R* = 0.71, *P* < .0001; IL-8 with IL-2: *R* = 0.81, *P* < .0001; MMP-2 with TIMP-1: *R* = 0.78, *P* < .0001; MCP-1 with IFN-γ: *R* = 0.70, *P* < .0001; IFN-γ with IL-2: *R* = 0.66, *P* < .0001. IFN-γ = interferon-gamma, IL-2 = interleukin-2, IL-8 = interleukin-8, MCP-1 = monocyte chemotactic protein-1, MMP-2 = matrix metalloproteinase-2, TIMP-1 = tissue inhibitor of metalloproteinases-1, TNF-α = tumor necrosis factor-alpha, VEGF = vascular endothelial growth factor.

The correlation between the expression levels of AH protein markers was also analyzed. There was a positive correlation between TNF-α and MCP-1, IFN-γ and IL-2, IL-8 and VEGF, IL-8 and IL-2, MMP-2 and TIMP-1, MCP-1 and IFN-γ, and IFN-γ and IL-2 (Figure 8. TNF-α with MCP-1: *R* = 0.76, *P* < .0001; TNF-α with IFN-γ: *R* = 0.92, *P* < .0001; TNF-α with IL-2: *R* = 0.65, *P* < .0001; IL-8 with VEGF: *R* = 0.71, *P* < .0001; IL-8 with IL-2: *R* = 0.81, *P* < .0001; MMP-2 with TIMP-1: *R* = 0.78, *P* < .0001; MCP-1 with IFN-γ: *R* = 0.70, *P* < .0001; IFN-γ with IL-2: *R* = 0.66, *P* < .0001.)

## 4. Discussion

The present study revealed that the AH levels of TNF-α, MMP-2, MCP-1, IFN-γ, TIMP-1, IL-6, IL-8, VEGF, and LT-α were different in patients with POAG, CACG, and APAC.

Cytokine networks in AH may play critical roles in the progression of APAC, CACG, and POAG. AH supplies nutrients and antioxidants to the cornea, lens, and TM.^[12,13]^ Thus, the differential expression of cytokines in the 3 types of glaucoma may help us to understand the various pathologies of APAC, CACG, and POAG. The levels of LT-α, BDNF, and PDGF-AA in the AH of different types of glaucoma have not been previously reported.

LT-α plays specific roles in the development and function of the immune system, the organization and maintenance of the lymphoid microenvironment, host defense, and inflammation. Compared with cataract eyes, we detected a significant decrease in LT-α levels in POAG eyes, whereas LT-α levels in APAC eyes were below the detection limits. Previous studies have shown that various inflammatory factors are significantly increased in the AH of patients with primary closed glaucoma, which is more obvious in eyes with APAC.^[14]^ LT-α is traditionally considered a proinflammatory cytokine; however, some studies have reached opposite conclusions. For example, a British study on fatigue symptoms central to a recognized proinflammatory mechanism found a negative correlation between blood LT-α levels and fatigue in patients with primary Sjogren syndrome.^[15]^ If LT-α has a dual role in promoting and suppressing inflammation, this would seem to explain our results. Therefore, we speculated that LT-α may not only promote inflammation, as suggested by most studies, but also have more complex mechanisms. We also found that the temporal and the lower quadrant RNFL were significantly correlated with the expression level of LT-α, suggesting that LT-α may have a neuroprotective effect on retinal ganglion cells (RGC).

BDNF levels are typically measured in patients with neurological disorders.^[16]^ Although BDNF expression in the eye has been explored in numerous experimental studies, data on BDNF levels in the AH of patients with glaucoma are limited. There were no significant differences in BDNF levels between the APAC and control groups, or between the CACG and control groups. The most exciting new finding of the present study was that BDNF levels were significantly reduced below the detection limits in the POAG group. It can be assumed that the POAG process is accompanied by or related to intraocular BDNF deficiency. The neuroprotective role of BDNF in the retinal ganglion has been demonstrated in glaucoma animal models.^[17,18]^ The role of BDNF in axon regeneration remains controversial.

PDGF-AA is a ligand for the PDGF receptor and a member of the receptor tyrosine kinase family.^[19]^ Previously, Chong et al identified PDGF-AA as a novel neuroprotective factor protecting RGC in retinal explant cultures and in a laser-induced ocular hypertension model of glaucoma.^[20,21]^ PDGF-AA may promote RGC survival through a direct effect on the PI3 kinase/AKT pathway, which prevents cell death.^[22]^ Compared with the CG, there was no difference among the three types of glaucoma. Our results may serve as a rationality for the clinical application of BDNF and PDGF-AA in glaucoma treatment.

VEGF, one of the most important inducers of angiogenesis and vascular permeability, also has a strong link with inflammation and immunity^[23,24]^ and can greatly change the tissue microenvironment and induce inflammation.^[25,26]^ Contrary to the sharp rise in VEGF levels in APAC eyes, VEGF remained at a slightly higher level in the CACG eyes, indicating a hypoxic microenvironment in the anterior segment tissues of the CACG and the APAC eyes. It has been postulated that VEGF expression correlate with glaucoma aggressiveness and refractory treatment. This cytokine is associated with multiple cytokine levels in neovascular glaucoma, suggesting it plays a role in promoting disease development.^[27]^ The levels of VEGF were in the same range as those reported in previous studies that utilized similar methods in patients with POAG.

In a previous study, the patients APAC had significantly higher levels of IL-6, IL-8, and MCP-1 in AH samples than cataract controls.^[14]^ We got the same conclusion in our study. These findings support the hypothesis that inflammation plays a key role in glaucoma pathogenesis. We also found that IL-6 levels were significantly correlated with CV, whereas IL-6 levels were significantly negatively correlated with HEX and CD levels. With regard to corneal endothelial morphology, CV appears to be the most sensitive of the 3 factors of corneal endothelial morphology. Therefore, IL-6 level may be a marker that determines the severity of corneal endothelial cell damage.

This study has some limitations. The use of topical antiglaucoma medications may influence the aqueous immune milieu. It is also possible that the chronic administration of prostaglandin eye drops influence or elevate the levels of inflammatory cytokines.^[28]^ Another limit of this study is the small sample size; the number of patients enrolled in our study was relatively small. However, the results were statistically significant. Therefore, the relatively small number of patients may have strengthened the results and conclusions of the present study. However, further studies with larger sample sizes would be helpful to explore and understand the precise mechanisms of these cytokines and the pathways involved in the pathogenesis of angle-closure glaucoma.

In summary, our study showed that the expression levels of TNF-α, IL-2, IL-6, IL-8, BDNF, MMP-2, MCP-1, VEGF, IFN-γ, LT-α, bFGF, PDGF-AA, and TIMP-1 were different among the 3 types of glaucoma. Different types of glaucoma may be caused by different pathogeneses, which opens avenues for further investigation into the pathogenesis of glaucoma and discoveries new targets and pathways for the treatment of glaucoma.
